# Plasma BDNF Levels Vary in Relation to Body Weight in Females

**DOI:** 10.1371/journal.pone.0039358

**Published:** 2012-07-02

**Authors:** Anilkumar Pillai, Davide Bruno, Antero S. Sarreal, Raymundo T. Hernando, Leslie A. Saint-Louis, Jay Nierenberg, Stephen D. Ginsberg, Nunzio Pomara, Pankaj D. Mehta, Henrik Zetterberg, Kaj Blennow, Peter F. Buckley

**Affiliations:** 1 Department of Psychiatry and Health Behavior, Medical College of Georgia, Georgia Health Sciences University, Augusta, Georgia, United States of America; 2 Division of Geriatric Psychiatry, Nathan S. Kline Institute for Psychiatric Research, Orangeburg, New York, United States of America; 3 Corinthian Diagnostic Radiology, New York, New York, United States of America; 4 Center for Dementia Research, Nathan S. Kline Institute for Psychiatric Research, Orangeburg, New York, United States of America; 5 New York University School of Medicine, New York, New York, United States of America; 6 New York State Institute for Basic Research in Mental Retardation, Staten Island, New York, United States of America; 7 Sahlgrenska University Hospital, Göteborg, Sweden; Baylor College of Medicine, United States of America

## Abstract

Brain derived neurotrophic factor (BDNF) has been implicated in the pathophysiology of depression as well as neuropsychiatric and neurodegenerative disorders. Recent studies show a role of BDNF in energy metabolism and body weight regulation. We examined BDNF levels in plasma and cerebrospinal fluid (CSF) samples from age matched elderly depressed and control subjects. Also, the association of BDNF levels with age, gender, body weight, body mass index (BMI), and cognitive performance was evaluated. We did not find any significant differences in plasma and CSF BDNF levels between depressed and control subjects. Plasma BDNF levels were negatively correlated with age (but not with BMI and body weight), when analyses were performed including both depressed and control subjects. A significant reduction in plasma BDNF levels was observed in females as compared to male subjects, and the change in BDNF levels were significantly and positively related to body weight in females. Furthermore, significant increases in Total Recall and Delayed Recall values were found in females as compared to males. In conclusion, the lower BDNF levels observed in females suggest that changes in peripheral BDNF levels are likely secondary to an altered energy balance. However, further studies using larger sample size are warranted.

## Introduction

Brain derived neurotrophic factor (BDNF) has been implicated in many neuronal functions including survival, neurogenesis, and synaptic plasticity [1]–[2]. BDNF regulates the development and plasticity of neural circuits involved in mood disorders such as depression [3]. *In vitro* studies show that BDNF stimulates the growth of dendrites and increases the spine density of cortical pyramidal neurons [4]–[5]. Exposure to stress and glucocorticoids has been shown to reduce BDNF expression in several brain regions including prefrontal cortex [6]–[8]. Moreover, postmortem brain studies have shown decreased levels of BDNF in prefrontal cortex in individuals who were depressed at the time of death [9]–[10]. Alterations in BDNF protein levels were also found in peripheral samples from depressed subjects [11]. A major source of the peripheral BDNF is platelets, which bind, store, and release BDNF upon activation [12].

Recent studies suggest that BDNF plays an important role in regulating energy homeostasis and body weight [13]–[15]. Studies in animals have shown that mice with only one functional BDNF allele exhibited a tendency toward obesity [16]. It is also known that eripheral injections of BDNF produce hypophagic and hypoglycemic effects in obese hyperglycemic animals, indicating antiobesity and antidiabetic effects [17]–[19]. Moreover, BDNF and its cognate receptor, TrkB are expressed in various hypothalamic nuclei implicated in the regulation of eating behavior [16]. In clinical studies, decreased levels of serum BDNF have been found in underweight women with anorexia nervosa [20]. In addition, a recent meta-analysis confirmed a reciprocal link between depression and obesity in humans [21]. However, the relationship between BDNF and body weight in depression is not clear.

In the present study, we compared BDNF levels in plasma and cerebrospinal fluid (CSF) samples from subjects diagnosed with major depressive disorder (MDD) and matched control subjects, and explored possible relationships between BDNF levels and factors such as age, gender, body mass index (BMI) or body weight. Since BDNF plays an important role in cognitive function, we examined the association of plasma BDNF levels with cognitive measures.

## Results

### Plasma and CSF BDNF Levels in Depressed and Control Subjects

BDNF levels as measured by ELISA did not show any significant difference between control and MDD subjects in plasma and CSF samples (Table 1). Furthermore, within the depression group, BDNF levels in subjects who are actively depressed and those in remission did not differ from levels in control subjects (Table 2). We did not find any significant effect of antidepressant medications on plasma and CSF BDNF levels in depressed subjects as compared to depressed subjects without antidepressants or control subjects (data not shown).

**Table 1:** BDNF levels in plasma and CSF samples from controls and MDD subjects.

Controls N = 43	Depressed N = 45	Controls N = 18	Depressed N = 29
plasma			CSF
210.12 (103.556)	225.21 (127.03)	7.75 (3.08)	6.57 (3.35)

BDNF values (pg/ml) are expressed as mean (± standard deviation).

**Table 2:** Effect of severity in depressive state on BDNF levels in plasma and CSF samples.

	plasma		CSF	
	BDNF (pg/ml)	N	BDNF (pg/ml)	N
controls	210.12 (103.55)	43	7.75 (3.08)	18
remission	248.90 (117.55)	17	6.32 (2.51)	12
Actively depressed	210.84 (132.45)	28	6.75 (3.91)	17

BDNF values (pg/ml) are expressed as mean (standard deviation).

### Effects of Age, BMI, Gender and Body Weight on BDNF Levels

**Figure 1:**
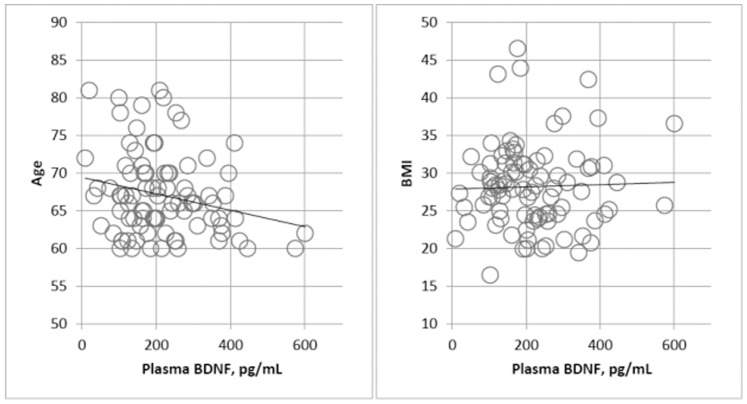
Association between plasma BDNF levels and age or BMI.

A negative correlation was found between age and plasma BDNF levels, when the analysis was done with depressed and control subjects together [r = −0.230, p = 0.031; Fig 1a). However, plasma BDNF did not show any significant correlation with BMI [r = 0.031, p = 0.774; Fig 1b) or body weight [r = 0.118, p = 0.277]. The correlation analysis was also performed in depressed and control subjects separately. No significant correlation was found between the above variables and plasma BDNF or CSF BDNF levels in each diagnostic group [r = 0.259, p = 0.146]. To determine whether gender plays any significant role in BDNF levels, plasma as well as CSF BDNF levels were examined in males and females. We found a significant decrease in plasma BDNF levels in females as compared to males (p = 0.044; Table 3). However, no significant change in BDNF levels was found in CSF samples between females and males.

**Table 3:** Effect of gender on plasma BDNF levels.

	gender	N	BDNF(pg/ml)
BDNF plasma	Male	44	244.94 (122.82)
	Female	48	195.26 (105.52)*
BDNF CSF	Male	25	6.71 (3.2)
	Female	22	7.38 (3.38)

BDNF values (pg/ml) are expressed as mean (standard deviation).

* p = 0.044.

Since BDNF has been implicated in the regulation of food intake and body weight we examined the relationship between BDNF and body weight in our study subjects. We found a significant reduction in body weight in females as compared to males (162.61 lbs ±31.74 vs. 202.99±117.73; p = 0.009). Moreover, when weight was considered as a covariate in ANCOVA, we did not find any significant difference in plasma BDNF levels between male and female subjects (p = 0.095) indicating a possible role of weight in the observed changes in plasma BDNF levels. When BMI was considered as a covariate in the above analysis the difference between genders in plasma BDNF levels remained significant (p = 0.046). However, we were not able to do the analysis with age as a covariate as the assumptions were not met to perform the analysis.

### Cognitive Performance

Since BDNF plays an important role in cognitive function, we next examined the association of plasma BDNF levels with cognitive measures. In depressed subjects, we found a significant positive correlation of plasma BDNF levels with Delayed recall (r = 0.366, p = 0.015, N = 44), Boston Naming Test (BNT; r = 0.307, p = 0.043, N = 44) and Letter fluency (r = 0.443, p = 0.003, N = 44). In control subjects, plasma BDNF levels significantly and negatively correlated with Total Recall (r = −0.314, p = 0.04, N = 43) and Digit Symbol scores (r = −0.383, p = 0.011, N = 43). No correlation between plasma BDNF and Hamilton Depression Rating Scale (HamD) scores was found in control or depressed group.

Next we examined whether the lower BDNF levels observed in females reflect in their cognitive performance. We did not find any significant difference in Wechsler Adult Intelligence Scale-vocabulary (Wais Voc), Elements, Digit Symbol and BNT measurements between males and females (Table 4). However, significant increases (p<.001) in Total Recall and Delayed Recall values were found in females as compared to males indicating a better cognitive performance in these two memory tasks in females.

**Table 4:** Cognitive performance measurements.

	gender	N	values
Wais Voc	Male	53	12.79 (2.99)
	Female	65	13.72 (2.38)
Total Rec	Male	53	69.18 (13.13)
	Female	65	59.02 (13.29)*
Elements	Male	53	1.88 (1.71)
	Female	65	1.86 (1.56)
Digit Sym	Male	51	12.31 (2.56)
	Female	64	12.96 (2.62)
Del Rec	Male	53	7.67 (2.81)
	Female	65	9.52 (2.87)*
BNT	Male	53	26.90 (3.60)
	Female	65	27.20 (2.03)

Values are expressed as mean (standard deviation).

* p<0.001.

### Relation between Plasma and CSF BDNF Levels

We did not find any significant correlation between plasma and CSF BDNF levels, when the analysis was done with depressed and control subjects together (data not shown). However, plasma BDNF levels showed a significant positive correlation with CSF BDNF levels in depressed subjects (r = 0.551, p = 0.014, n = 19), but not in controls. In addition, a significant positive correlation was found between plasma and CSF BDNF levels in females (r = 0.601, p = 0.030; N = 13), but not in males, in all subjects.

## Discussion

Although BDNF has been well studied for its roles in neuroprotection and neuroplasticity, recent studies suggest that BDNF is also involved in energy homeostasis and metabolic functions. Our results revealed a significant effect of body weight on plasma BDNF levels in females. Also, plasma BDNF levels were significantly lower in females indicating gender specific alterations in circulating BDNF levels.

Our study did not find any change in plasma or CSF BDNF levels in depressed subjects as compared to controls. In addition, no significant difference in BDNF levels was found between subjects who are actively depressed and those in remission, indicating a lack of plasma BDNF relationship with the severity of depression. Our observations are not in agreement with many of the previous findings from preclinical and clinical studies. In particular, decreases in BDNF levels have been reported in animal models of depression [22] and BDNF administration has been shown to produce antidepressant effects [23]–[25]. In addition, general physical activity [26] and chronic antidepressant treatment [27] have been shown to up-regulate BDNF mRNA levels in the rat hippocampus. Data from clinical studies have reported reductions in plasma BDNF levels in depressed subjects [28]–[30]. Moreover, peripheral BDNF levels showed negative correlations with the severity of depression in many studies [31]–[33] with the exception of a few reports showing no significant correlations [34]–[35]. The discrepancy between our study and other reports could be due to many factors such as time of blood withdrawal, time of storage, food intake before sampling, urbanicity, age, sex, smoking status and drinking behavior [36]–[37]. In addition, the present study examined BDNF levels in the plasma rather than serum samples from patients and control subjects. It is known that serum BDNF largely reflects the amount of BDNF stored in circulating platelets, whereas plasma BDNF may reflect to a larger extent the steady-state situation and consequently the amount of bioactive protein [38]. Most of the studies, which found significant differences in the BDNF levels between depressed and control subjects have used either platelets or serum samples for BDNF analysis [28].

An important finding from our study is that plasma BDNF levels show positive correlations with body weight in all the subjects, but not in each diagnostic group. BDNF infusion into the rat brain has been shown to lower body weight and to suppress appetite [39]–[40]. In addition, heterozygous BDNF mice exhibit progressive obesity [41]. The above rodent studies are supported by clinical evidence that polymorphisms in the BDNF gene are significantly associated with eating disorders in humans [42]–[44]. In addition, BDNF levels in plasma have been shown to decrease significantly with increasing age or weight in healthy human subjects [45]. These studies suggest an inverse relationship between BDNF and body weight. According to the above assumptions, we should find an increase in BDNF levels in females as they have lower body weight as compared to males in our study. Therefore, the decrease in plasma BDNF levels in females observed in our study suggests that changes in peripheral BDNF levels are likely secondary to an altered energy balance. Consistently, a positive association between peripheral BDNF and body weight has been found in women with obesity [46]. Alternatively, peripheral and brain BDNF levels may be modulated by different regulatory elements which require further follow up in MDD and control subjects as well as relevant animal models.

Stress and glucocorticoid hormones are known to impair cognitive function [47]. Moreover, studies from BDNF heterozygous knockouts and knockdowns revealed impairments in cognitive functions [48] indicating a potential role of BDNF in cognitive function. Although plasma BDNF levels were lower in female subjects in our study, these subjects displayed better cognitive performance in the Total Recall and Delayed Recall tests. In this regard, BDNF levels have been shown to correlate negatively with a number of working memory tasks in aged rats, but not young rats [49]. It is also important to note that peripheral BDNF levels as determined by routine ELISA methods are poor in differentiating proBDNF and mature BDNF levels. Moreover, studies have indicated that proBDNF and mature BDNF peptides elicit opposite cellular responses through the receptors, p75^NTR^ and TrkB, respectively [50]. Therefore, additional studies using isoform specific BDNF antibodies as well as additional measurements on cognitive performance are warranted to confirm the role of peripheral and brain-derived BNDF in cognitive functions.

Since significant changes in plasma BDNF levels were found only in females, one can speculate that the alterations in plasma BDNF levels were related to changes in the secretion of estrogens. Estrogen has been shown to regulate the expression of BDNF, and estrogen and BDNF have overlapping actions in the brain [51]–[52] **.** Moreover, postmenopausal women exhibit more depressive behavior [53] and high levels of estrogen enhance associative memory formation in ovariectomized animals [54]. The average age of female subjects in our study is 66.15+/?5.3 years. There is mounting evidence that TrkB is downregulated in specific brain regions (including CA1 pyramidal neurons and cholinergic basal forebrain neurons) in mild cognitive impairment [55]–[56] and peripheral ganglia during the normal aging process [57]–[58]. Further studies exploring the relationships between circulating estrogens and BDNF in the above study subjects would help to confirm or disregard the role of estrogens in the regulation of plasma BDNF levels.

In the present study, we observed a significant positive correlation between plasma and CSF on BDNF levels in depressed subjects. Few studies have investigated BDNF levels in CSF, although it has the promise of being the bodily fluid of choice for biomarker studies to determine brain-specific molecular alterations and pathological abnormalities [59]. A recent study has demonstrated a strong relationship between plasma and CSF BDNF levels in first episode psychosis subjects [60].

Our study has the following limitations. The number of CSF samples in our study was lower as compared to plasma samples and therefore, the correlation analysis between plasma and CSF on BDNF levels was performed only in a small number of matched samples. Similarly, we were unable to determine the cortisol levels in plasma samples. Such data would have been helpful to evaluate the possible role of cortisol in the regulation of BDNF levels in our study.

In conclusion, the present study reports that plasma BDNF levels are not different in depressed subjects as compared to controls, and are not related to the severity of depression. In addition, plasma BDNF levels are lower in females, and body weight shows a positive association with BDNF levels in these subjects. Further assessment of these observations in separate, larger cohorts is warranted.

## Materials and Methods

### Ethics

This study was approved by the Institutional Review Boards of both the Nathan Kline Institute for Psychiatric Research and the NYU School of Medicine. Participants were all volunteers who responded to advertisements in local newspapers and flyers. All subjects provided formal consent before being examined and were compensated up to $450.

### Subjects

Demographic characteristics of the participants are shown in Table 5. The total number of 133 participants completed the baseline visit; out of these 133 subjects, a total of 14 participants were excluded because of evidence in the MRI of confluent deep or periventricular white matter hyperintensities, defined as one or more hyperintense lesions measuring at least 10 mm in any direction, or because of a Mini-Mental State Exam (MMSE) score below 28. Out of these 119 remaining subjects, 47 individuals took part in the optional lumbar puncture procedure in which a CSF sample for determination of various neurobiological markers, including cortisol and BDNF, was collected. Of these 47 subjects, 28 were diagnosed with MDD based on clinical evaluation and SCID for DSM IV, and 19 were controls. Plasma for determination of BDNF levels was available for 88 subjects out of 119; out of these 88 individuals, 45 had a diagnosis of MDD and 43 did not.

**Table 5:** Case demographics.

	Controls N = 43	Depressed N = 45	T-test	Controls N = 19	Depressed N = 28	T-test
	plasma				CSF	
Age, in years	67.0 (5.3)	67.1 (5.7)	t(86) = 0.036, p = 0.971	68.1 (7.3)	66.5 (5.4)	t(45) = 0.835, p = 0.408
Education in years	16.6 (2.6)	16.2 (2.9)	t(85) = 0.716, p = 0.476	16.7 (2.7)	16.5 (2.7)	t(44) = 0.274, p = 0.785 a
BMI	27.2 (4.6)	29.3 (6.3)	t(86) = 1.776, p = 0.079	28.1 (4.7)	28.8 (6.7)	t(45) = 0.378, p = 0.707
MMSE	29.7 (0.5)	29.8 (0.6)	t(86) = 0.695, p = 0.489	29.5 (0.5)	29.8 (0.6)	t(45) = 1.56, p = 0.126

Values are expressed as mean (± standard deviation).

BMI  =  Body-mass index;

MMSE  =  Mini-mental State Exam score;

a =  one subject’s years of education was not available.

### Cognitive Tests

Subjects also took part in a neuropsychological evaluation, which included the following cognitive tests: Vocabulary Subtest of the WAIS-R [61], Digit-Symbol Substitution subtest of the WAIS-R, Letter Fluency [62], Category Fluency [63], Boston Naming Test [64], and the following components of the Buschke Selective Reminding Task: Total Recall score across seven learning trials, Delayed Recall after 20 minutes, and Elements defined as the number of unique incorrect words produced during a recall task (BSRT) [65]–[66].

### Procedure

This study was conducted over four visits, generally each 1 week apart. The first three visits were conducted at the Nathan Kline Institute, Orangeburg, NY and at the Clinical & Translational Science Institute, NYU Langone Medical Center. During the first visit, participants were explained the study procedures and provided informed consent. Also medical and psychiatric history, and vital signs were obtained at this stage. Subjects also underwent a psychiatric evaluation, and global cognitive status was assessed using the MMSE. During visit 2, participants received an MRI scan of the head to quantify the magnitude of vascular brain pathology. During the third visit, participants underwent a comprehensive neuropsychological assessment. During visit 4, for a subset of our subjects, a lumbar puncture (LP) was performed by a neuroradiologist. Subjects were required to fast overnight prior to the lumbar puncture visit, which was performed between 9–10 AM. After fasting, a total of 15 ml of clear CSF were collected, centrifuged at 4 degrees C at 1500 rpm for 10 minutes, and stored at –80 degrees C. For those subjects who did the lumbar punctures, blood for BDNF assay was drawn in the morning, under fasting conditions and immediately prior to the LP (Visit 4). For individuals who chose not to have the LP, blood for BDNF was also drawn at approximately the same time in the morning and under fasting conditions, but during the baseline visit (visit 3). Thus, any correlations between plasma and CSF BDNF levels pertain to samples drawn at approximately the same time in the morning and on the same day. Blood samples were centrifuged at 3000 rpm for 30 minutes at 4°C. Plasma was carefully collected and was stored at −80°C, until used for further analyses.

### BDNF Assay

Plasma levels of BDNF were determined with an enzyme-linked immunosorbent assay (ELISA) method (BDNF Emax Immunoassay System, Promega, USA), according to the manufacturer’s instructions as described previously [57]. Briefly, 96-well flat bottom immunoplates were coated with an anti-BDNF monoclonal antibody and incubated at 4°C overnight. After blocking by non-specific binding with Block & Sample Buffer, standards and samples were added to the plates and incubated and shaken for 2 h at room temperature. Subsequently, after washing with TBST wash buffer, plates were incubated for 2 h with Anti-Human BDNF polyclonal antibody. The last incubation required the addition of Anti-immunoglobulin Y-horse-radish peroxidase conjugate. In the last step of the assay, TMB One solution was added in order to develop the color. After stopping the reaction with HCl 1 N, the absorbance was read at 450 nm on a microplate reader and BDNF concentrations were determined automatically according to the BDNF standard curve (ranging from 7.8 to 500 pg ml^−1^ purified BDNF). The sensitivity of the assay was 4 pg/ml. All the samples were analyzed in duplicate in one session by an investigator blind to experimental set up.

### Statistical Analysis

Bivariate correlations (Pearson’s r tests), t-tests and Analyses of Variance (with control variables; ANCOVA) were performed. All tests were two-tailed and statistical significance was judged at α = .05. All analyses were conducted in SPSS 15.0 or PASW 18.0.
